# The potential of a targeted unilateral compound training program to reduce lower limb strength asymmetry and increase performance: a proof-of-concept in basketball

**DOI:** 10.3389/fphys.2024.1361719

**Published:** 2024-06-26

**Authors:** Wenfeng Zhang, Xing Chen, Kun Xu, Hezhi Xie, Jiayong Chen, Ziwei Zhu, Hongshen Ji, Duanying Li, Jian Sun

**Affiliations:** ^1^ School of Athletic Training, Guangzhou Sport University, Guangzhou, China; ^2^ Guangdong Key Laboratory of Human Movement Function Science, School of Athletic Training, Guangzhou Sport University, Guangzhou, China; ^3^ Sihui High School-High-Tech School, Sihui, China; ^4^ Graduate School, Guangzhou Sport University, Guangzhou, China

**Keywords:** sports performance, strength and conditioning, between-limb, imbalance, power

## Abstract

**Objective:**

This study investigates the efficacy of training methodologies aimed at mitigating asymmetries in lower limb strength and explosiveness among basketball players.

**Methods:**

Thirty male university basketball athletes were enrolled in this research. Initial assessments were made regarding their physical attributes, strength, and explosiveness. Subsequently, the participants were randomly allocated into two groups: an experimental group (EG, *n* = 15) and a control group (CG, *n* = 15). Over 10 weeks, the EG engaged in a unilateral compound training regimen, incorporating resistance training exercises such as split squats, Bulgarian split squats, box step-ups, and single-leg calf raises (non-dominant leg: three sets of six repetitions; dominant leg: one set of six repetitions) and plyometric drills including lunge jumps, single-leg hops with back foot raise, single-leg lateral jumps, and single-leg continuous hopping (non-dominant leg: three sets of 12 repetitions; dominant leg: one set of 12 repetitions). The CG continued with their standard training routine. Assessments of limb asymmetry and athletic performance were conducted before and after the intervention to evaluate changes.

**Results:**

1) Body morphology assessments showed limb length and circumference discrepancies of less than 3 cm. The initial average asymmetry percentages in the single-leg countermovement jump (SLCMJ) for jump height, power, and impulse were 15.56%, 12.4%, and 4.48%, respectively. 2) Post-intervention, the EG demonstrated a significant reduction in the asymmetry percentages of SLCMJ height and power (*p* < 0.01), along with improvements in the isometric mid-thigh pull (IMTP) test metrics (*p* < 0.05). 3) The EG also showed marked enhancements in the double-leg countermovement jump (CMJ) and standing long jump (SLJ) outcomes compared to the CG (*p* < 0.01), as well as in squat performance (*p* < 0.05).

**Conclusion:**

The 10-week unilateral compound training program effectively reduced the asymmetry in lower limb strength and explosiveness among elite male university basketball players, contributing to increased maximal strength and explosiveness.

## 1 Introduction

Basketball, a highly popular sport classified as an invasion game, has been extensively studied over the past decade (Stojanovic et al., 2018). Basketball research has focused on identifying performance indicators (Sergio et al., 2018), technical–tactical aspects (Canan and Hirata, 2019; Fernández-Cortes et al., 2021), health (Shao and Sun, 2022), and load control (Piñar et al., 2022), among others. In the area of health, one specific term has experienced exponential growth in recent years: asymmetries.

Inter-limb asymmetries, a focal point of recent research, refer to the comparative analysis of the functionality between one limb and its counterpart (Keeley and Oliver, 2011). Between-limb imbalance in strength and power, assessed as the limb symmetry index, has been considered a valid and useful tool to detect players at high risk (e.g., 4-fold in players with >10% asymmetry) of lower extremity injury (Gustavsson et al., 2006). Additionally, inter-limb asymmetries might also play a role in performance (e.g., more symmetrical team-sports players seem to be faster than their asymmetrical counterparts) (Bailey et al., 2013; Lockie et al., 2014). Less research has been conducted on asymmetries in basketball than in other invasion sports, such as soccer (Nunes et al., 2018; Buoite Stella et al., 2022).

Research analyzing asymmetries in basketball compares the differences obtained among groups in test batteries. These test batteries are essentially composed of two types of tests: laboratory tests, in which flexion–extension is measured through peak torque (Schiltz et al., 2009a; Parpa and Michaelides, 2022), and field tests, in which straight runs and vertical jumps with one or two legs are used (Bakaraki et al., 2021; Barrera-Domínguez et al., 2021). Theoharopoulos and Tsitskaris (2000) and Rahnama and Bambaecichi (2005) suggest that reaching a certain level of expertise in basketball can lead to lower limb strength and flexibility asymmetries. Schiltz et al. (2009b) examined isokinetic knee extensor and flexor strength in professional and junior basketball players to determine the presence of lower limb explosive strength asymmetry and its differences. The results showed that isokinetic and functional variables were similar between groups, with no dominant differences, but basketball players with knee injuries exhibited bilateral isokinetic strength asymmetry. Radjo et al. (2013) measured morphological and force indicators in basketball players to determine the degree of differences between the two main components (left leg and right leg) of the basketball player’s movement system. They measured morphological and kinetic indicators in 68 basketball players using the Biodex isokinetic system and balance system, and statistical analysis revealed significant differences. Leroy et al. (2000) compared the spatial and temporal gait variables of 10 swimmers, 10 basketball players, and 16 soccer athletes (all men) using a gait analysis system, observing differences in gait patterns between the left and right sides. The basketball and soccer players exhibited asymmetric gait variables, while swimmers did not show statistically significant differences in gait variables between the left and right sides, suggesting differences across different sports. Basketball is considered a symmetrical sport, meaning that there are no inherent differences in asymmetries caused by training, competition, various tasks, game situations, or specific player positions (Sergio et al., 2023). Any natural movement asymmetries that may arise during the sport are typically compensated for by subsequent movements. However, there may be isolated instances where certain players exhibit asymmetries that are not directly related to basketball practice. In such cases, it is crucial for the coaching staff to promptly identify the asymmetry and implement corrective measures to minimize or eliminate it. Although it is generally accepted that minimizing the differences between the two sides of the body is logical and that reducing asymmetry on both sides of the body is beneficial for human movement, there is little research on how to reduce the degree of asymmetry between two sides of a body. In this regard, strength and explosive training is one of the most used strategies for reducing asymmetry (Blakeyl and Southard, 1987; Lundin and Berg, 1991; Adams et al., 1992; Huerta et al., 2016). The term “complex training” specifically refers to arranging rapid, plyometric exercises similar in biomechanical nature to resistance training immediately following the resistance training within the same session (Ebben WPWP. and Watts P. B., 1998; Carter and Greenwood, 2014). Newton and Kraemer (1994) describe complex training as a training strategy involving explosive muscle actions and integrating rapid and slow force outputs. They suggest that this training method can simultaneously enhance both strength and speed, implying an increase in maximal explosive force. In early literature, when describing the combination of strength training with plyometric exercises, terms such as “combination lifts in the complex” and “mixed-method training” were used instead of “complex training.” Over time, these terms gradually evolved into “complex training” (SKK, 1986; Newton and Kraemer, 1994; Batista et al., 2011). Additionally, to the authors’ knowledge, no studies have yet analyzed the effects of interventions on inter-limb asymmetry among basketball players; therefore, further research in this demographic is warranted.

This study aims to evaluate the impact of unilateral compound training on limb asymmetry among male basketball players and to determine whether alterations in limb asymmetry influence overall physical performance. It is posited that the unilateral compound training intervention will lead to significant enhancements in both limb asymmetry and physical performance from pre-intervention to post-intervention.

## 2 Methods

### 2.1 Experimental approach to the problem

**FIGURE 1:**
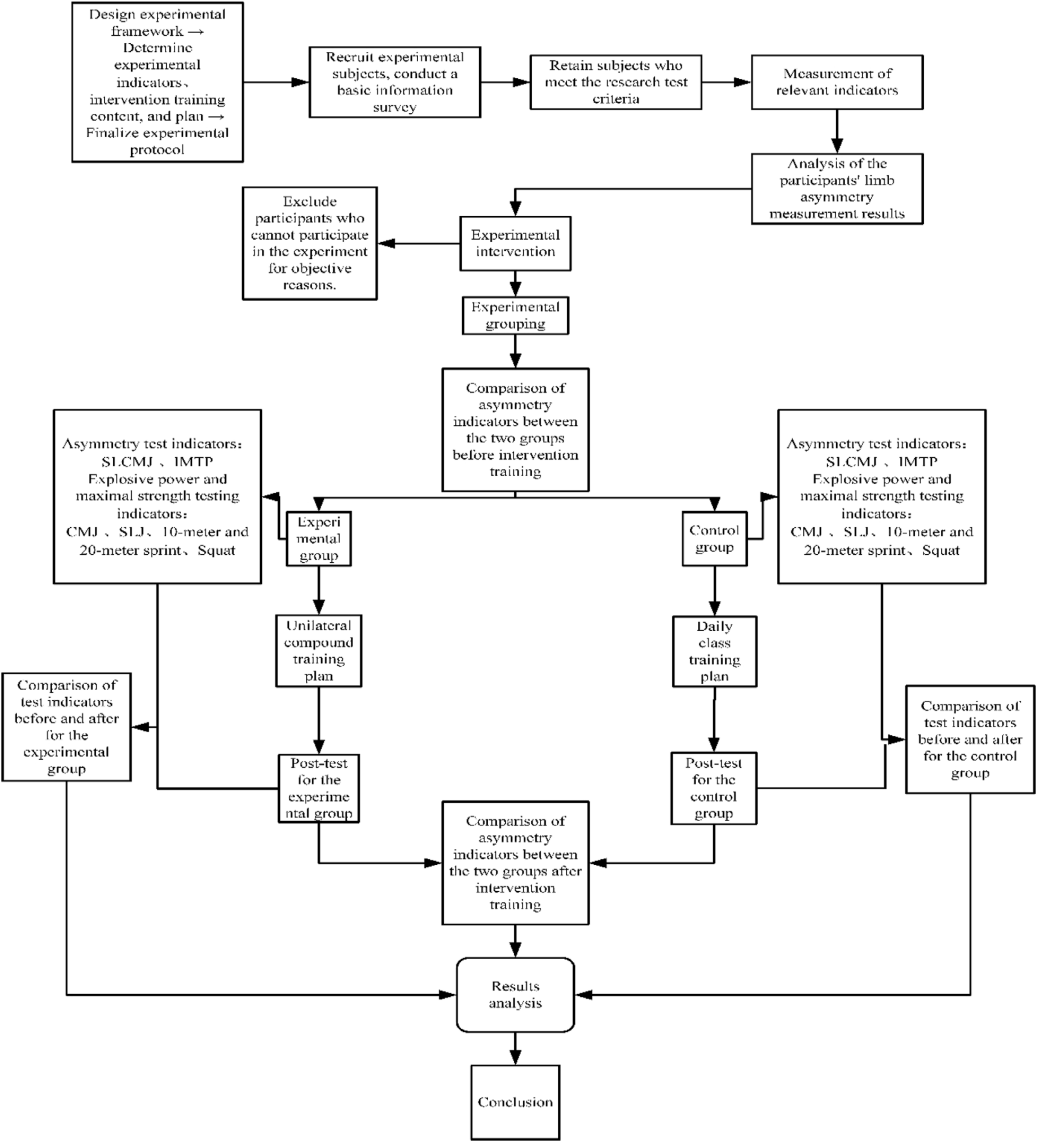
Flowchart of the experiment.

Before the intervention, participants’ body morphology (length and girth) and the asymmetry index of lower limb explosive power (SLCMJ) were measured to assess the degree of asymmetry. It was hypothesized that asymmetry would exist in both limb morphology and strength among the participants. Additionally, to further assess the asymmetry in lower limb force-generating capacity, IMTP testing was conducted using a portable force plate (400 Series Performance Force Plate, Fitness Technology, Adelaide, Australia) sampling at 600 Hz, along with a portable IMTP rack (Fitness Technology, Adelaide, Australia). This approach enables a more objective and accurate detection of the strength differences between the two sides of the lower limbs, thereby providing a comprehensive reflection of inter-limb strength disparities. Subsequently, the study employed a single-factor, completely randomized, pretest–posttest research design. Participants underwent unilateral compound training aimed at improving limb asymmetry. Our second hypothesis posited that this intervention would reduce the percentage difference in limb asymmetry among participants. Participants were randomly assigned to either an experimental group (EG) (*n* = 15) or a control group (CG) (*n* = 15). The EG underwent unilateral compound training, characterized by a relatively uniform intensity and volume of the training load. The intervention lasted between 4 and 10 weeks and included one to six sessions of resistance training and five to 15 sessions of plyometric training per week. Each session was controlled to include two to five sets at a frequency of 1 to 3 times per week (Haff, 2016; Michael, 2016). Rest intervals should be appropriately tailored to accommodate individual responses to the compound training regimen. The unilateral training regimen adheres to the principles of Michael Boyle’s unilateral functional training approach (Michael, 2016). The specific intervention protocol for this study is detailed in Table 1. The intervention spanned 10 weeks, occurring three times per week (Monday, Wednesday, and Friday), with each session lasting 30 min before class ended, followed by a unified cool-down exercise lasting 5–10 min. The CG maintained their usual training regimen. Subsequent analyses compared pre- and post-training changes in test indicators to evaluate whether improvements in limb asymmetry influenced sports performance levels. This led to the formulation of a third hypothesis: that reducing limb asymmetry positively affects sports performance. All participants were fully briefed on the associated benefits, risks, measurement protocols, and procedures and participated in a standardized familiarization session prior to testing. A standardized warm-up, which included joint mobility exercises, dynamic stretching, mid-zone activation, and specific exercises such as weighted half squats and sprints, was conducted before the official tests (Beato and Coratella, 2021). Experienced investigators provided on-site technical feedback. If an athlete’s movements were not executed correctly or lacked full effort, the tests and training sessions were required to be repeated. The experimental procedure for this study is illustrated in Figure 1.

**TABLE 1:** Experimental intervention plan.

Grouping	Training methods	Training content	Number of sets/repetitions	Load intensity	Rest interval	Total duration (min)
_EG_	Resistance training	1. Split squat	Non-dominant leg: three sets of six repetitions (3 × 6)	Resistance training and plyometric compound training overcome body weight, requiring participants to exert maximum effort to complete the movements	40 s	13
	Plyometrics	2. Bulgarian split squat	Dominant leg: one set of six repetitions (1 × 6)		5 min	5
		3. Box step-up	Non-dominant leg: three sets of 12 repetitions (3 × 12)		60 s	12
		4. Single-leg calf raise	Dominant leg: one set of 12 repetitions (1 × 12)			
		1. Lunge jump				
		2. Single-leg hop with back foot raise				
		3. Single-leg lateral jump				
		4. Single-leg continuous hopping				
_CG_	Regular course content					

Note: Load intensity is to overcome their own weight.

### 2.2 Specific intervention plan

#### 2.2.1 Participants

Thirty elite male university basketball players voluntarily participated in the study. The inclusion criteria were the absence of injuries or illnesses as confirmed by the Physical Activity Readiness Questionnaire (Thomas and Shephard, 1992); right-handedness with right-hand dominance; left leg as the predominant leg for basketball activities, notably as the takeoff leg in a three-step layup maneuver. Participants regularly engaged in basketball team training, consisting of three 120-min basketball sessions (Monday, Wednesday, and Friday) and one physical training session (Saturday) per week, had over a year of experience in heavy strength resistance training, and were health-screened by two physical training experts with an average of 12 years experience in physical training, testing, and evaluation. The athletes typically performed resistance training weekly in the strength and conditioning lab as part of their training regimen and were familiar with the training protocol and testing methods. Prior to the trial, all participants were informed of the potential risks and benefits and signed an informed consent form. They were instructed to maintain their usual exercise routine 48 h before the trial and to abstain from any stimulants or alcohol. The final analysis included 30 participants (age 20.9 ± 1.0 years; weight 71.3 ± 6.3 kg; height 180.4 ± 5.2 cm; training years 4.1 ± 0.8 years; body fat percentage 18.4% ± 4.2%) (Table 2). This study received approval from the Academic Ethics Committee (2023LCLL-68), and all procedures were conducted in accordance with the Declaration of Helsinki for human participants (Association, 2013).

**TABLE 2:** Baseline characteristic.

Variable	EG (N = 15)	CG (N = 15)	Total	*P*	*ES*
Age (years)	20.9 ± 1.1	20.9 ± 0.9	20.9 ± 1.0	0.91	0.01
Body Mass (kg)	73.5 ± 5.3	69.1 ± 6.7	71.3 ± 6.3	0.56	0.73
Height (cm)	182.1 ± 4.1	178.7 ± 5.8	180.4 ± 5.2	0.78	0.68
Body mass index (kg/m^2^)	16.2 ± 4.6	14.3 ± 2.5	18.4 ± 4.2	0.32	0.51

#### 2.2.2 Procedures

##### 2.2.2.1 Measure

###### 2.2.2.1.1 Inbody370 body composition

Inbody370 Body^®^ Composition Analyzer Usage: Athletes were instructed to remove their shoes and socks and stand on the analyzer’s electrode plates. They input their ID, height, age, gender, and other details into the display screen and held the measurement handles on either side, placing their thumbs on the bipolar plates. Their arms should rest naturally at their sides until the measurement is complete. The system generated a test report from which relevant indicators were selected based on the experimental needs. The data were stored on a computer for future reference, and comprehensive result reports, including nutrition and exercise plans, were available for print.

###### 2.2.2.1.2 Bilateral isometric mid-thigh pull

Bilateral IMTP testing followed similar protocols used in previous research (Thomas et al., 2015). The IMTP testing was performed on a portable force plate sampling at 600 Hz (400 Series Performance Force Plate, Fitness Technology, Adelaide, Australia) using a portable IMTP rack (Fitness Technology, Adelaide, Australia). Sampling as low as 500 Hz has been shown to produce high-reliability measures for isometric force-time variables (Dos Santos et al., 2016). The force plate was interfaced with computer software [Ballistic Measurement System (BMS)] that allowed direct measurement of force-time characteristics. For the bilateral stance IMTP testing, a collarless steel bar was positioned to correspond to the athlete’s second-pull power clean position just below the crease of the hip (Haff et al., 2015). The bar height could be adjusted in 3 cm increments at various heights above the force plate to accommodate different-sized athletes. Athletes were strapped to the bar in accordance with previous research (Haff et al., 2005) and positioned in their self-selected mid-thigh clean position established in the familiarization trials whereby feet were shoulder width apart, knees were flexed over the toes, shoulders were just behind the bar, and torso was upright (Dos’ Santos et al., 2016). Researchers have demonstrated that differences in knee and hip joint angles during the IMTP do not influence kinetic variables (Haff et al., 2005; Comfort et al., 2014), justifying the self-selected preferred mid-thigh position. All subjects received standardized instructions to pull as fast and as hard as possible and push their feet into the force plate until they were told to stop, as these instructions have been shown to be optimal in producing maximum PF and RFD results. IMTP assessments demonstrated high within-session reliability for PF (Thomas et al., 2015). Once the body was stabilized (verified by watching the subject and force trace), the IMTP was initiated with the countdown “3, 2, one pull,” with participants ensuring that maximal effort was applied for 5 s based on previous protocols (Haff et al., 2005; Haff et al., 2015). Data were collected for a duration of 8 s. Minimal pre-tension was allowed to ensure there was no slack in the body prior to initiation of pull. Verbal encouragement was given for all trials and subjects. Participants performed a total of three bilateral maximal effort trials interspersed with 2-min recoveries.

###### 2.2.2.1.3 Vertical jump tests

Single-leg countermovement jump (SLCMJ) procedure: Participants positioned themselves at the center of the Smart Jump mat with their hands on their hips. Upon receiving the initial command, they balanced on one leg, maintaining an upright posture for 1–2 s. A subsequent command prompted participants to perform a squat immediately followed by a maximal vertical leap, exerting full effort while continuing to balance on the same leg. During the aerial phase of the jump, it was crucial to maintain a vertically aligned torso. Upon landing—touching down with both feet—participants were required to execute knee flexion to absorb the shock. They then maintained a single-leg stance for an additional 1–2 s. A practice jump was performed prior to the official testing. Each movement was repeated three times per side with a 10-s rest interval between jumps, and a 1-min rest was allowed when switching sides. The highest recorded jump from each side was considered the valid test value. Double-leg countermovement jump (DLCMJ) procedure: The DLCMJ protocol differed only in that both takeoff and landing involved the use of both legs simultaneously.

The system used the formula peak power output (PPO) (W) = 60.7jump height (cm) +45.3mass (kg) −2055. The PPO was an estimate and not a measurement. The formula (flight time/1000) × (body weight) × (g/2) was used to calculate the impulse (N·s) of the athlete’s vertical jump, and the formula jump height (cm) = (flight time/1000) × (2g) × (100/8), where the unit of flight time was (ms), and the constant for gravity (g) = 9.81 m/s. The day before the test, the participants were briefed on the testing process and the standard movements of a stationary squat jump. The participants practiced to familiarize themselves with the key points of the stationary squat jump movement.

###### 2.2.2.1.4 Standing long jump tests

The participants positioned themselves comfortably with both feet entirely behind the takeoff line. Initiating the jump directly without preliminary movements such as stepping or hopping was mandatory. The distance was precisely measured from the takeoff line to the nearest point of first contact upon landing. Each participant executed three jumps, and the maximum distance achieved was recorded.

###### 2.2.2.1.5 10- and 20-meter straight sprint tests

The Brower Timing System (TC-1H, United States of America), a wireless apparatus that obviates the need for transmission lines, is capable of timing long distances and facilitating shuttle runs and agility tests. This system, enhanced with additional sensors, allows for subdividing a race start into multiple stages for granular analysis. It was strategically positioned at both the starting and finish lines of the 20 m sprint track, which was constructed from plastic material. Competitors were positioned less than 0.5 m from the starting line, in a high starting posture with feet spread, arms at their sides, and hips and knees moderately bent. Participants commenced the sprint at their discretion to eliminate variability in reaction times affecting the results. Timing began as participants crossed the initial photocell gate and ended upon completion at the finish line. This method provides a precise evaluation of sprinting capabilities independent of initial reaction times. The sprint durations for distances of 10 m and 20 m were meticulously recorded as 0.00 s. Upon arrival at the laboratory, participants engaged in a standardized warm-up, followed by a 3-min passive rest. The data from two sprint rounds of 20 m—with rest intervals based on participant rotation—were collected; the better performance was used as the benchmark for subsequent training sessions.

###### 2.2.2.1.6 Maximum strength tests

Before conducting the maximum strength test, it was necessary to estimate the participants’ one repetition maximum (1RM) weight, which should have been close to their maximum strength but not so heavy that they were unable to complete the movement. The testing procedure was as follows: First, the participants performed 10 warm-up reps with an empty barbell and then rested for 2–3 min. Second, the weight was increased by approximately 15% of the estimated 1RM for one set of 3–5 reps. The participants rested for 3–5 min and continued to increase the weight by 15% of the estimated 1RM, and so on. Third, once the weight reached 90% of the estimated 1RM, only a 5% increase for 1–2 reps was made, followed by a 5-min rest. Fourth, the weight was increased to the estimated 1RM for a trial lift; if successful, they rested for 5 min and then continued to increase by 5%; if unsuccessful, they rested for 5 min and attempted a second trial lift; if it failed again, they rested for 5 min and decreased the weight by 2.5%–5% for a trial lift. The participants were to determine their 1RM value within five attempts (Baechle, 2008).

###### 2.2.2.1.7 Statistical analyses

Statistical analysis was performed on SPSS software^®^ (v24.0, Chicago, United States). Normality and equal variance assumptions were checked using the Shapiro–Wilk test and Levene test, respectively. Statistical significance was inferred from *p* < 0.05. The percentage formula for unilateral asymmetry testing is (Dominant limb (DL) − Non-dominant limb (NDL))/DL × 100 (Nunn and Mayhew, 1988), and the percentage formula for bilateral asymmetry testing is (DL − NDL)/(DL + NDL) × 100 (Kobayashi et al., 2013). The data comparison between the two sides of the limbs was done using an independent *t*-test. Inter-group comparisons of various indicators were made using independent T-tests, while intra-group comparisons used paired T-tests. To control the pre-test variable, the pre-test results were treated as equal groups, and post-intervention comparisons of the test indicators in both groups were made using a one-way analysis of covariance (ANCOVA). During the covariance analysis, the post-test was set as the dependent variable, the pre-test as the covariate, and the group as the independent variable. In the one-way ANCOVA, data that did not meet the assumptions were analyzed using a *t*-test. The effect size in the covariance analysis was measured according to Cohen’s d effect value standards (Fritz and Richler, 2012). Partial η^2 values are small effect (≥0.01 and <0.06), medium effect (≥0.06 and <0.14), and large effect (≥0.14).

## 3 Results

### 3.1 Pre-intervention limb asymmetry measurements for participants

Body morphology and strength metrics underwent direct measurement and evaluation, adhering to the standards outlined in the textbook *Human Movement Ability Testing and Evaluation* (Li Jie, 2005).

The analysis revealed no significant differences in the body morphology metrics among the participants (*p* > 0.05). Research categorizes the degree of variation as mild (<3 cm), moderate (3 cm ≤ X < 6 cm), and severe (>6 cm) (Sayers and Bishop, 2017). Observation of the participants’ body morphology indicators suggests relative symmetry, with discrepancies between the left and right sides of the limbs consistently below 3 cm (Table 3).

**TABLE 3:** Participant body morphology measurement results (N = 30).

Variable (cm)	M ± SD	T	P
Upper limb length (left)	73.98 ± 1.84	−0.43	0.67
Upper limb length (right)	73.99 ± 1.85		
Upper arm length (left)	30.39 ± 1.08	−1.37	0.18
Upper arm length (right)	30.41 ± 1.07		
Forearm length (left)	22.29 ± 1.04	0.34	0.72
Forearm length (right)	22.34 ± 1.06		
Upper arm circumference (left)	29.36 ± 1.27	−1.96	0.06
Upper arm circumference (right)	29.46 ± 1.29		
Forearm circumference (left)	25.54 ± 1.09	−1.90	0.07
Forearm circumference (right)	25.60 ± 1.07		
Thigh length (left)	50.46 ± 1.19	1.69	0.10
Thigh length (right)	50.42 ± 1.17		
Calf length (left)	47.13 ± 1.11	−0.98	0.34
Calf length (right)	47.17 ± 1.17		
Lower limb length (left)	101.07 ± 2.58	1.07	0.29
Lower limb length (right)	101.04 ± 2.51		
Thigh circumference (left)	53.43 ± 2.36	−1.42	0.17
Thigh circumference (right)	53.29 ± 2.40		
Calf circumference (left)	29.73 ± 1.11	0.38	0.71
Calf circumference (right)	29.72 ± 1.06		
Ankle circumference (left)	20.86 ± 0.89	0.90	0.38
Ankle circumference (right)	20.84 ± 0.89		
Foot length (left)	27.36 ± 1.01	0.11	0.92
Foot length (right)	27.32 ± 0.98		

### 3.2 Pre-intervention SLCMJ test results

**FIGURE 2:**
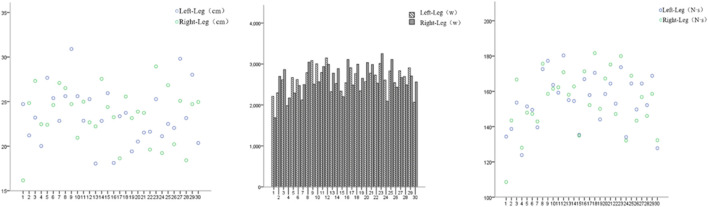
SLCMJ test metrics: cm = height, W = power, N·s = impulse.

SLCMJ test results, illustrated in Figure 2, demonstrate disparities between the left and right conditions concerning participants’ jump height, peak power, and impulse, with noticeable inconsistencies on both sides. Specifically, the average jump height for the 30 participants’ left and right legs was 25.42 cm and 23.50 cm, respectively; the average peak power was 2621.83 W for the left and 2657.25 W for the right; and the average impulse was 154.65 N·s for the left and 154.41 N·s for the right (see Table 4).

**TABLE 4:** SLCMJ test metrics (N = 30).

Variable	Limb	M ± SD	Degree of asymmetry%
SLCMJ (cm)	Left leg	25.42 ± 3.15	15.56 ± 7.77
	Right leg	23.50 ± 3.06	
Power (w)	Left leg	2621.83 ± 303.62	12.14 ± 6.11
	Right leg	2657.25 ± 354.12	
Impulse (N·s)	Left leg	154.65 ± 14.87	4.48 ± 3.88
	Right leg	154.41 ± 16.99	

Note: The calculation method for the asymmetry percentage of unilateral test indicators is (DL – NDL)/DL × 100.

### 3.3 The impact of unilateral compound training on the intervention of limb asymmetry

#### 3.3.1 SLCMJ test metric

Results on left-right asymmetry from the vertical jump mat SLCMJ tests are summarized in Table 5. For SLCMJ height, a significant post-experiment asymmetry was noted between the two groups (*p* < 0.01). Similarly, a significant difference in SLCMJ peak power asymmetry was observed post-experiment (*p* < 0.01). Conversely, for the SLCMJ impulse indicator, the asymmetry level did not differ significantly between the groups post-experiment (*p* > 0.05). Notably, the EG exhibited a lower asymmetry percentage of 3.01% than the CG’s 5.38%, indicating superior performance by the EG.

**TABLE 5:** Asymmetry percentages in SLCMJ tests before and after the experiment.

Variable (%)	Time	EG (N = 15)	CG (N = 15)	Comparison between two groups		Before and after the EG		Before and after the CG	
				T	P	T	P	T	P
Jump height	Pre	15.25 ± 5.68	15.86 ± 9.63	0.21	0.84	6.35	0.001^**^	1.49	0.16
	Post	7.86 ± 2.58	13.48 ± 5.46	3.60	0.002^**^				
Power	Pre	11.57 ± 5.99	12.70 ± 6.39	0.50	0.62	3.90	0.002^**^	1.08	0.30
	Post	5.15 ± 1.41	11.52 ± 6.60	3.66	0.002^**^				
Impulse	Pre	4.20 ± 1.43	4.75 ± 5.38	0.39	0.70	4.35	0.001^**^	−1.13	0.28
	Post	3.01 ± 1.21	5.38 ± 4.96	1.80	0.08				

Note: The calculation method for the asymmetry percentage of unilateral test indicators is (DL − NDL)/DLD × 100; *significantly different from two groups at *p* < 0.05; ** significantly different from two groups at *p* < 0.01.

#### 3.3.2 Isometric mid-thigh pull

The results from the isometric leg strength test asymmetry between the left and right legs are detailed in Table 6. Post-experiment, a significant asymmetry difference was observed between the two groups (*p* < 0.05), with the EG demonstrating 3.20% asymmetry and the CG demonstrating 10.20%. The post-experiment asymmetry levels increased by 0.2% in the CG and decreased by 5.87% in the EG. Post-experiment asymmetry for the EG was reduced to 3.20% from its pre-experiment level of 9.07%.

**TABLE 6:** Asymmetry percentages in isometric lower limb strength tests.

Variable (%)	Time	EG (N = 15)	CG (N = 15)	Comparison between two groups		Before and after the EG		Before and after the CG	
				T	P	T	P	T	P
Isometric mid-thigh pull (PF)	Pre	9.07 ± 5.12	10.00 ± 7.13	0.41	0.68	−5.44	0.000^**^	0.14	0.89
	Post	3.20 ± 2.11	10.20 ± 2.95	7.48	0.001^**^				

Note: The asymmetry percentage for bilateral test indicators is calculated as (DL − NDL)/(DL + NDL) × 100; PF: peak force.

#### 3.3.3 Intervention outcomes on participants’ athletic performance results

##### 3.3.3.1 Explosive power (EP) and maximum strength (MS) variables

Table 7 shows a comparison of variables between the two groups pre-experiment. Table 8 shows an analysis of Covariance Assumptions for Participants’ EP and MS Variable. Post-experiment analyses using covariance for CMJ and SLJ heights are detailed in Table 9, revealing F (1,28) = 8.73, η^2^_*p* = 0.24 for CMJ and F (1,28) = 11.98, η^2^_*p* = 0.31 for SLJ. Controlling for baseline measures, significant differences in CMJ and SLJ heights were found between the experimental group (EG) and the control group (CG) (*p* < 0.05). An independent *t*-test for the 20 m sprint indicated no significant group differences (*p* > 0.05), with an effect size (ES) of 0.63, suggesting a medium effect; specifically, the post-test 20 m sprint speeds were 2.87 s for the EG and 2.97 s for the CG, demonstrating faster performance by the EG. The EG’s growth rate of −0.02 surpassed the CG’s rate of −0.01. Regarding squat performance, covariance analysis post-intervention (Table 9) showed F (1,28) = 4.86, η^2^_*p* = 0.15, with significant intergroup differences post-control (*p* < 0.05) and a substantial effect size. The EG’s squat performance (117.76 kg) exceeded the CG’s (117.37 kg), with growth rates of 0.05 for the EG and 0.04 for the CG, as detailed in Table 10.

**TABLE 7:** Comparison of participant EP and MS variables before the experiment.

Variable	CG (N = 15)	EG (N = 15)	T	P
CMJ (cm)	49.90 ± 4.63	53.43 ± 5.21	−1.96	0.06
SLJ (cm)	273.87 ± 11.60	279.07 ± 13.31	−1.14	0.26
10-m sprint (s)	1.81 ± 0.06	1.81 ± 0.09	0.07	0.95
20-m sprint (s)	3.02 ± 0.10	2.93 ± 0.19	1.6	0.12
Squat (kg)	113.83 ± 14.07	111.50 ± 15.75	0.43	0.67

**TABLE 8:** Analysis of covariance assumptions for participant EP and MS variables.

Hypothesis Variable	Linear hypothesis		Homogeneity of variance test		Parallel assumption interaction term *p*-value	
	F	P	F	P	F	P
CMJ (cm)	30.54	0.001^**^	2.29	0.09	0.97	0.33
SLJ (cm)	64.76	0.001^**^	2.44	0.13	0.005	0.95
10-m sprint (s)	2.14	0.12	0.10	0.75	0.02	0.90
20-m sprint (s)	27.77	0.000^**^	2.01	0.17	6.41	0.02
Squat (kg)	174.09	0.001	0.07	0.79	0.03	0.86

Note: If the *p*-value for the linear hypothesis is less than 0.05, it meets the condition for covariance analysis; if the *p*-value for the homogeneity of variance test is greater than 0.05, it meets the condition for covariance analysis; if the *p*-value for the parallelism assumption is greater than 0.05, it meets the condition for covariance analysis. A *t*-test was conducted for factors not meeting the assumption conditions in one-way ANCOVA.

**TABLE 9:** Results of the covariance analysis for participants’ EP and MS tests.

Variable	CG (N = 15)	EG (N = 15)	F	P	η^2^_p
CMJ (cm)	51.37^a^ ± 0.81	54.86^a^ ± 0.81	8.73	0.006**	0.24
SLJ (cm)	275.25^a^ ± 1.14	280.88^a^ ± 1.14	11.98	0.002**	0.31
10-m sprint (s)	1.74^a^±0.02	1.70^a^±0.02	3.42	0.08	0.11
Squat (kg)	117.37^a^ ± 0.84	117.76^a^ ± 0.84	4.86	0.04	0.15

Note: “a” represents the adjusted mean ± standard deviation of the dependent variable after covariate correction. In the covariance analysis, a *t*-test is conducted for conditions that do not meet the assumptions.

**TABLE 10:** Comparison of changes in participant EP and MS test results.

Variable	CG d% (N = 15)	EG d% (N = 15)	T	P
CMJ (cm)	0.01 ± 0.07	0.06 ± 0.05	−2.64	0.01
SLJ (cm)	−0.002 ± 0.02	0.01 ± 0.01	−2.66	0.01
10-m sprint(s)	−0.04 ± 0.04	−0.06 ± 0.05	1.35	0.19
20-m sprint(s)	−0.01 ± 0.04	−0.02 ± 0.02	0.62	0.54
Squat (kg)	0.04 ± 0.03	0.05 ± 0.02	−0.23	0.82

Note: d = (post-test measurement − pre-test measurement)/pre-test measurement × 100.

## 4 Discussion

This study aimed to assess the effects of a unilateral compound training regimen on limb asymmetry and to explore its impact on physical performance among male basketball players. The principal outcomes indicate that this training regimen significantly decreased limb asymmetry and that reductions in asymmetry correlated positively with enhanced explosive power and maximal strength parameters. Consequently, this evidence substantiates the integration of strength and explosive training into basketball training protocols.

The examination and comparison of morphological indicators between the participants’ left and right limbs revealed that discrepancies in length and circumference were minimal (less than 3 cm), and these differences were not statistically significant. These results are consistent with those of previous research findings (Beattie et al., 1990; Rhodes et al., 1995). In basketball training, players’ skills, such as dribbling and ball handling with both hands, can promote balanced development on both sides of the body (Sergio et al., 2023). Studies have shown that shorter limbs can bear higher peak loads and higher loading rates than longer limbs. In the long term, individuals with slightly different lower limb lengths may be more prone to muscle and skeletal problems due to greater forces and loads applied to the shorter limb. Therefore, individuals with lower limb length differences ranging from 1 cm to 3 cm should also consider achieving balanced limb lengths (White and Wilk, 2004). Bell et al. (2014) found that the asymmetry in thigh and calf circumference explained 25% of the variance in reactive strength measures, and the asymmetry in pelvic, thigh, and calf lean mass explained 25% of the variance in lower limb explosive power, such as countermovement jumps. Hence, differences in lean body mass between limbs may partly cause strength and power asymmetry and could potentially limit jump height optimization when considering their impact (Boyi et al., 2014).

Limb strength asymmetry directly influences strength and power performance, which in turn affects various other athletic skills and overall sports performance. Newton et al. (2006) measured the limb imbalance through bilateral and unilateral squats of equal length and found that the lower limb strength difference was 1% in the group with accurate kicks, while the group with less accurate kicks showed differences exceeding 8%. This indicates that higher lower limb strength asymmetry has a negative impact on the accuracy of kicking in athletes (Hart et al., 2014). Bazyler et al. (2014) also demonstrated that higher limb asymmetry reduces vertical jump height and maximal explosive power. Research on elite cyclists showed a negative correlation between the asymmetry of peak torque at the knee joint (180°/sec) and power output during a 5-s maximal cycling test (r = −0.50; *p* < 0.05). Asymmetry in lower limb explosive power affects body movement, direction changes, regulation of the body’s center of gravity, the execution of specialized techniques, agility, multidirectional speed, and more. Maloney investigated the correlation between asymmetry in single-leg hopping and a 90° cutting task. The study divided participants into fast and slow groups, and the asymmetry in average vertical stiffness and vertical jump height explained 63% of the cutting performance (r = 0.63; *p* = 0.001). Additionally, the faster group of athletes had lower asymmetry in jump height, indicating that reducing asymmetry in lower limb jumping can effectively enhance cutting performance (Maloney and Richards, 2016). Lockie et al. (2014) investigated athletes exhibiting varying degrees of lower limb explosive power asymmetry through multiple assessments, including vertical jumps, short-distance sprints, and agility tests. The findings indicated that moderate asymmetry in lower limb explosive power during jumping did not significantly correlate with performance in short-distance sprinting or agility. However, excessive asymmetry adversely impacted enhancements in sprinting and agility performance.

The study utilized unilateral compound intervention training techniques to diminish limb asymmetry and concurrently enhance maximal strength and explosive power in lower limb squats. This aligns with previous research findings by Maloney and Richards (2016), Brown et al. (2017), and Bishop and Read (2018), who consider that intervention training can reduce inter-limb asymmetry and enhance physical performance. A recent meta-analysis by Bettariga et al. (2022) investigated the effects of training interventions on inter-limb asymmetries measured across a range of physical performance tests. In summary, the asymmetry tests most used to demonstrate changes in side-to-side differences are a range of unilateral jump and change of direction (COD) speed tests. When training methods are considered, most traditional resistance programs have utilized a combination of strength and jumping-based exercises over 6–10 weeks. The rationale behind this is that resistance training stimulates the secretion of anabolic hormones, activating skeletal muscle protein synthesis, promoting muscle fiber hypertrophy, and enhancing muscle strength and explosive power (Damas and Ugrinowitsch, 2018; Gallo-Villegas et al., 2018; Grgic et al., 2018). However, resistance training, typically performed slowly, lacks intense neural stimulation and only increases muscle explosive power by enhancing maximal strength. In contrast, plyometric training utilizes the lengthening–shortening cycle, activating the stretch reflex and storing elastic potential energy in muscles for more forceful contractions (Ebben WPWPB. and Watts P. B., 1998). Explosive power, being the product of strength and speed, benefits from the combined advantages of plyometric training, effectively exercising both strength and speed (Robert and William, 1994).

During unilateral training, the central nervous system and proprioceptors activate a comprehensive array of muscle groups, potentially utilizing the unique physiological process known as “cross-education.” Governed primarily by neural pathways, including those in the cerebral cortex and spinal cord, this phenomenon’s impact varies with the training approach. Notably, while this study employed bilateral limb interventions, it disproportionately focused on the weaker limb through increased training sessions and sets, indicating that the effects of cross-education are significant (Beobachtungen, 1858; Scripture and Brown, 1894). Future investigations should intensively explore the mechanisms behind cross-education to enhance its practical application. Furthermore, evidence suggests that unilateral training significantly boosts strength, explosive power, and agility. Derrick-E Speirs et al. (2016) investigated the impact of 5 weeks of unilateral *versus* bilateral squat training on strength, short-distance sprints, and multidirectional speed, aiming to delineate the comparative benefits of these training modalities on athletic performance. Appleby et al. (2019) allocated 33 athletes into three groups: a unilateral group, a bilateral group, and a CG. They engaged in lower limb strength training twice weekly, with the bilateral group performing squat exercises and the unilateral group undertaking weight-bearing single-leg push-offs from a box. The findings indicated that both training modalities enhanced maximum lower limb strength, short-distance sprint capability, and multidirectional speed; however, unilateral training showed a more pronounced effect on multidirectional speed. Furthermore, unilateral training was associated with greater activation of the gluteus medius, enhanced knee joint stability, and a reduced immediate testosterone response than bilateral training. However, a formal power analysis was not conducted during the research design phase to determine sample size. Given the exploratory nature of this study and its reliance on a small, specialized cohort, effective power calculations were not feasible. Future studies will incorporate power calculations to establish appropriate sample sizes.

In summary, unilateral training, with its high demands for limb instability and extensive muscle recruitment during training, effectively reduces inter-limb differences. Future research should integrate limb asymmetry assessment with basic strength and explosive power evaluations in basketball training practices. This approach would optimize the assessment system for basketball players, providing new tools to understand their physical deficiencies. Longitudinal studies to explore the long-term characteristics and trends of limb asymmetry in basketball players and its cyclical impact on their performance are suggested.

## 5 Conclusion

The limb length and circumference asymmetries in the measurements of the limbs in male college basketball players were less than 3 cm; however, the differences in strength and explosive power metrics between the limbs were more pronounced. A 10-week unilateral compound training intervention effectively reduced the percentage of asymmetry in these strength and explosive power metrics among male college basketball players. This reduction in limb asymmetry percentage difference positively impacted the associated metrics of maximal strength and explosive power.

## 6 Practical applications

In future basketball training practices, coaches must closely monitor the asymmetry and severity of asymmetry between the upper and lower limbs on both sides of athletes’ bodies. For athletes exhibiting significant asymmetry, targeted and specialized training should be implemented to reduce the asymmetrical differences between their limbs, thereby enhancing performance and preventing sports injuries. Furthermore, the study utilized loads based on the athletes’ own body weight, allowing them to apply what they have learned directly on the court post-training or competition. It is crucial to actively adopt advanced training concepts and methodologies that promote the balanced development of athletes’ limbs, improve training efficiency and effectiveness, and ultimately boost their overall performance and achievements.

## Data Availability

The original contributions presented in the study are included in the article/Supplementary Material, further inquiries can be directed to the corresponding authors.
